# PMMA-Etching-Free Transfer of Wafer-scale Chemical Vapor Deposition Two-dimensional Atomic Crystal by a Water Soluble Polyvinyl Alcohol Polymer Method

**DOI:** 10.1038/srep33096

**Published:** 2016-09-12

**Authors:** Huynh Van Ngoc, Yongteng Qian, Suk Kil Han, Dae Joon Kang

**Affiliations:** 1 Department of Physics and Interdisciplinary Course of Physics and Chemistry, Sungkyunkwan University, 2066, Seobu-Ro, Jangan-Gu, Suwon, Gyeonggi-do 16419, Republic of Korea; 2 Teraleader Inc., 55-8, Techno-11ro, Yuseong-gu, Daejeon 34036, Republic of Korea

## Abstract

We have explored a facile technique to transfer large area 2-Dimensional (2D) materials grown by chemical vapor deposition method onto various substrates by adding a water-soluble Polyvinyl Alcohol (PVA) layer between the polymethyl-methacrylate (PMMA) and the 2D material film. This technique not only allows the effective transfer to an arbitrary target substrate with a high degree of freedom, but also avoids PMMA etching thereby maintaining the high quality of the transferred 2D materials with minimum contamination. We applied this method to transfer various 2D materials grown on different rigid substrates of general interest, such as graphene on copper foil, h-BN on platinum and MoS_2_ on SiO_2_/Si. This facile transfer technique has great potential for future research towards the application of 2D materials in high performance optical, mechanical and electronic devices.

Two-dimensional (2D) materials have attracted much attention due to their unique properties and great potential in various applications. Controllable synthesis of 2D materials with high quality and high efficiency is essential for their large scale device applications. Chemical vapor deposition (CVD) has been one of the most reliable technique for the synthesis of 2D materials. To explore novel applications and the discovery of new phenomena in these materials, it is necessary to develop a process to transfer high quality and large area 2D materials onto desirable substrates with great efficiency and high yield. Currently, the most commonly used transfer method relies on the use of sacrificial polymethyl-methacrylate (PMMA) film to support the 2D layers and to prevent them from folding during the etching of the growth substrate. However, the removal of PMMA is usually incomplete and leads to surface contamination due to undissolved PMMA residues^1^,^2^,^3^,^4^,^5^,^6^,^7^. These contaminants are trapped on the surface of the transferred 2D materials, resulting in the degradation of the intrinsic properties, and reduce the reliability of devices fabricated from them. This effect also has consequences for the formation of multiple layer heterostructures from 2D materials in that it severely limits future work on these, which are usually formed using layer-by-layer transfer methods. Other transfer methods which do not involve PMMA have been also investigated, including using thermal release tapes as transfer membranes^1^, direct transfer onto polydimethylsiloxane (PDMS)^2^, or using two-layer structures consisting of polyethylene terephthalate (PET) and silicone^3^, and hot pressing^4^. But so far, the wide applicability of these methods is still limited due to limitations in size, uniformity and quality of the transferred 2D layers. Moreover, eliminating the metal etching step and transferring graphene by a direct delamination from the growth substrate is the most effective way to avoid contamination from both metal and PMMA impurities. Several direct delamination and transfer methods without PMMA have been also proposed, such as, use of mechanical fracture testing instrument^5^,^6^ and Polyvinyl Alcohol (PVA) assisted transferring^7^. However, the degree of freedom and uniformity in the transfer process is still significantly limited by graphene not being in an isolated state, since the delamination and the transfer of graphene are performed simultaneously using an adhesion layer. Moreover, wrinkling of the graphene surface due to the Poisson effect of the elastomer stamp during the transfer process cannot be avoided. Especially, direct delamination methods to transfer 2D transition metal dichalcogenide materials grown on oxide substrates have not been demonstrated due to their strong Van der Waals interaction between the 2D transition metal dichalcogenide materials and its growth oxide substrates.

In this communication, we demonstrate that adding a water soluble PVA layer in-between the PMMA layer and 2D material grown on a rigid substrate allows not only the effective transfer to arbitrary target substrates with a high degree of freedom but also avoids the etching of the PMMA layer to obtain contamination-free high quality 2D materials by avoiding detrimental effects related to surface contaminants. Graphene transferred by this process exhibits excellent quality, indicated by a charge neutrality point being close to zero. Due to the elimination of contamination from the PMMA residues, both graphene and MoS_2_ FETs fabricated using our transfer method showed higher mobility and current modulation values compared to those formed using conventional PMMA assisted transfer of the 2D materials. We describe the details of the PMMA/PVA based transfer method for 2D materials grown on different rigid substrates, for instance, graphene on copper foil, h-BN on platinum and MoS_2_ on SiO_2_/Si substrates. The quality of the transferred 2D materials has been evaluated systematically and the versatility of the transfer method is demonstrated by fabricating reliable graphene and MoS_2_ field effect transistors.

## Results and Discussion

**Figure 1:**
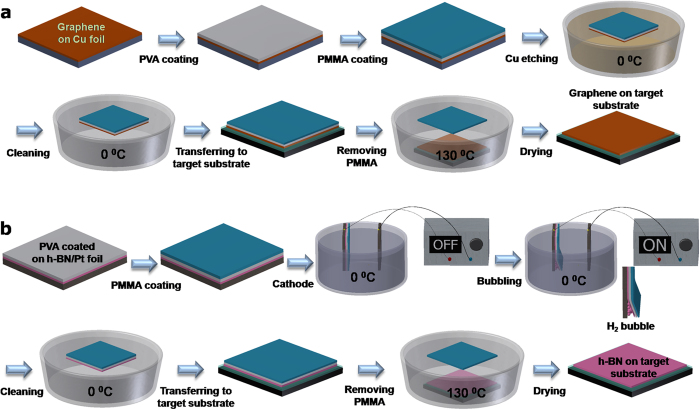
Schematic illustration of PVA assisted transfer of CVD-grown 2D materials. (**a**) Graphene transfer by copper etching method, (**b**) Single layer h-BN transfer by bubbling method.

Figures 1a,b show schematic illustrations of the PMMA/PVA assisted transfer of CVD graphene and h-BN, respectively, onto various rigid target substrates. The detailed transfer procedure is described in the Methods Section. There are three essential steps in the PMMA/PVA assisted transfer of graphene: first, coating PMMA/PVA onto the graphene, second, etching of the Cu layer and third, releasing the graphene layer followed by the transfer onto the target substrate. The crucial breakthrough in our newly developed PMMA/PVA assisted method when compared with the conventional PMMA assisted method is in the third step. Usually, PMMA is removed by dissolving in acetone, whereas, in the PMMA/PVA assisted method, PMMA is released in hot water at 130 °C. Due to the low viscoelastic properties of PVA, PVA alone is not strong enough to support the transfer of graphene, thus it can easily break in a form of thin film. Therefore, PMMA is still needed as an intermediate supporting membrane in transferring 2D materials similar to the conventional PMMA assisted 2D materials transfer method, thus facilitating easy handling of 2D materials. On the other hand, in the PMMA/PVA assisted transfer method, a thin PVA layer of 100 nm is employed as a buffer layer between graphene and PMMA. As PVA can be easily dissolved in hot water, PMMA can be peeled off directly, leaving graphene on the substrate. We exploited the PVA’s unique property of being easily dissolved in hot water, thus leaving no trace on the surface of the 2D materials as demonstrated in the transfer of mechanically exfoliated 2D layers^8^. Therefore, with the PMMA/PVA assisted transfer method, which has all the advantages of the conventional PMMA transfer method such as simplicity, large area, and high degree of freedom, we can easily transfer 2D materials of different morphologies onto arbitrary target substrates with controlled transfer orientation while minimum contamination. There are a few studies that use a similar method for the crystal transfer^9^,^10^,^11^,^12^. However, it should be clearly noted that in this dry-transfer method, the 2D material is mechanically transferred onto the top of PMMA/PVA and 2D/PMMA layer is released from the substrate by dissolving PVA in water and then transferred to a target substrate for device fabrication. Therefore, the complete removal of PMMA from 2D material remains as a technical issue to be resolved.

**Figure 2:**
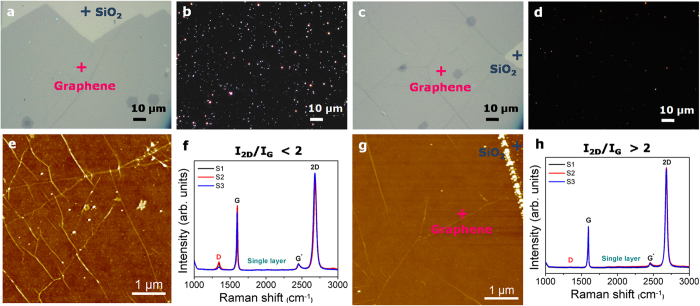
Graphene transfer. Optical microscopy images of (**a,c**) bright field and (**b,d**) dark field, (**e,g**) AFM images and (**f,h**) Raman spectra of transferred graphene on SiO_2_/Si substrate by copper etching method using PMMA and PVA, respectively.

We have compared the quality of the graphene layers transferred onto SiO_2_/Si substrate by PMMA and PMMA/PVA assisted transferring methods by using optical microscopy, atomic force microscopy (AFM), Raman spectroscopy and electrical measurement. Figure 2 shows optical microscopy (a,c) bright field and (b,d) dark field images of graphene on SiO_2_/Si substrate transferred by copper etching method using PMMA and PMMA/PVA, respectively. Compared to the PMMA method, the graphene transferred by PMMA/PVA exhibited a much cleaner surface. The problem associated with the incomplete etching of PMMA has been well documented; acetone cleaning cannot completely remove PMMA residues due to their strong Van der Waals interaction with graphene^13^,^14^,^15^. The morphological features of graphene transferred using both transfer methods were also examined using AFM (Fig. 2e,g). Graphene transferred using PMMA/PVA shows a flat and clean surface after transfer and no residual polymer particles are observed, in clear contrast to the graphene transferred using PMMA. Contamination-free high quality graphene layer was obtained simply by dissolving PVA in hot water, even without any additional thermal-annealing process^7^,^15^,^16^.

Raman spectroscopy is a quick and unambiguous method to evaluate the quality and the number of graphene layers. Figure 2f,h are representative Raman spectra of the transferred single-layer graphene on the target substrate (SiO_2_/Si) using the PMMA and PMMA/PVA assisted transfer methods, respectively. The difference of the D, G and 2D peaks indicates the quality and uniformity of the graphene^3^. The D peak, at 1,350 cm^–1^ is caused by the presence of structural disorder, randomly distributed impurities, surface charges or doping in graphene^7^. Disorder in sp^2^-hybridized carbon systems leads to resonance in their Raman spectra, which makes Raman spectroscopy one of the most sensitive techniques to characterize such disorder and to evaluate the quality of graphene^17^,^18^,^19^. Here we found that the high intensity of the D peak in the case of PMMA transferred sample is due to the presence of PMMA residues or due to wrinkles formed during the transfer process^20^,^21^. The D peak is almost absent in the case of the PMMA/PVA transferred graphene, which suggests that the graphene sustained physical damage and/or contamination both during the transfer processes and during PMMA/PVA removal, thus leading to uniform and high quality transferred graphene^17^,^18^. Moreover, it has been demonstrated that the increase in the 2D/G ratio points to a cleaner surface with lower doping level or contamination^22^,^23^. The Raman spectra also showed that the 2D/G ratio of the graphene transferred using PMMA/PVA is greater than 2 while the 2D/G ratio of graphene transferred by PMMA is less than 2, indicating a better quality and cleaner surface for the PMMA/PVA transferred graphene.

An additional thermal annealing was performed for graphene transferred by both PMMA and PMMA/PVA methods in an attempt to remove polymer residues on the surface and thereby reduce the number of trapping sites at the interface between the transferred graphene and the target SiO_2_/Si substrate^14^,^16^,^24^. The annealing was carried out at 250 °C for 6 h under a gas flow containing 100 sccm argon and 5% hydrogen. As shown in Supplementary Fig. S1, even after the thermal annealing process, some PMMA residues were still observed in the AFM images indicating the difficulty in completely removing these residues from the graphene surface. Thus, the difficulty to completely remove residual PMMA by annealing is the main drawback that leads to a high Dirac voltage and a large hysteretic window in FETs made from graphene transferred using PMMA^14^,^24^. The quality of the PMMA/PVA-transferred graphene on the SiO_2_/Si substrate after annealing, was also evaluated by Raman spectroscopy as shown in Supplementary Fig. S2. We observed the D band for the annealed graphene whereas we observe no D band before annealing (Fig. 2h). We therefore conclude that annealing at high temperature not only brings graphene in close contact with SiO_2_ dielectric layer that increases the coupling between them, but also introduces other undesirable defects, resulting in the activation of the D band. These two observations may lead to heavy hole doping and a severe degradation of carrier modulation and reduced mobility in graphene FET devices as reported by Cheng *et al*.^15^ Thus, taking into account the advantage of our PMMA/PVA transferring method, high quality, large area and clean 2D materials can be easily transferred even without the need for subsequent thermal treatment, which makes this process particularly attractive for the fabrication of various devices that need to be fabricated at low temperature. It is obvious that the scale of the graphene transferred by PMMA/PVA can be as large as that transferred by the conventional PMMA method, thus making it viable for large-area graphene transfer for industrial applications.

**Figure 3:**
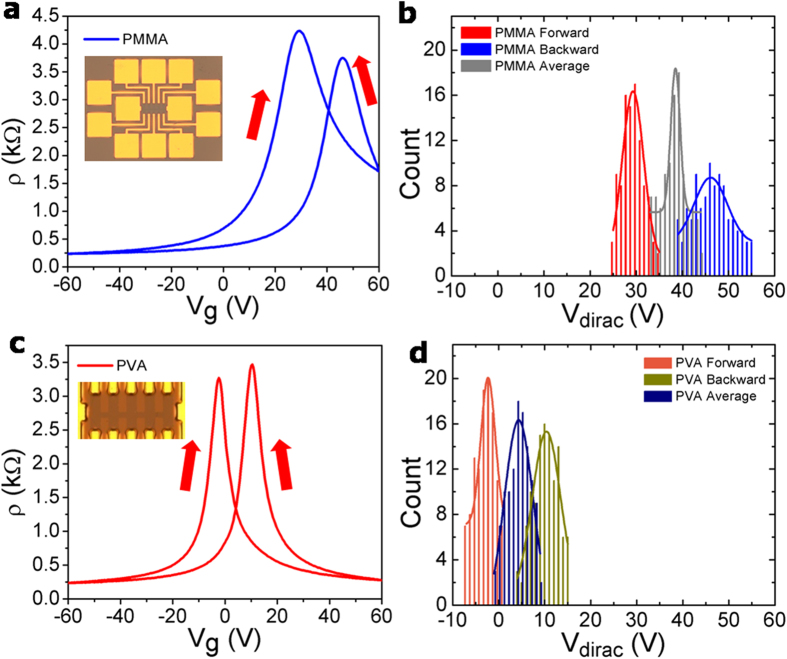
Graphene FETs characteristics. (**a,c**) Resistivity versus the back gate voltage, (**b,d**) Histogram of Dirac points, of graphene on SiO_2_/Si substrate transferred by copper etching method using PMMA and PVA, respectively. Insets show the optical images of the corresponding devices.

To evaluate the electrical property of the transferred graphene, back-gated graphene FETs were fabricated using graphene transferred on 100 nm thick SiO_2_/Si substrates, with Cr/Au (20/50 nm) as the source and drain electrodes, respectively. Figure 3 shows the electrical characteristics for graphene FETs in which graphene was transferred both by the conventional PMMA and by the newly developed PMMA/PVA methods. A drastic enhancement in the device performance was clearly observed in the PMMA/PVA transferred graphene FETs in terms of 1) Dirac voltage (*V*_Dirac_) and its distribution, 2) hysteresis, and 3) modulation of electron and hole currents. Figure 3a,c represent the resistivity versus the back gate voltage and Fig. 3b,d show the histograms of Dirac voltages of the graphene FETs transferred by the copper etching method with the help of PMMA and PMMA/PVA transfer methods while the gate voltage was scanned from −60 V to 60 V forward and backward. The optical images of the corresponding graphene FETs are shown as insets in the figures. We note that, a high Dirac voltage was observed for the PMMA transferred sample, which changed from 29 to 46 V when the gate voltage was swept forward and backward. The 17 V difference between the Dirac voltages for the forward and backward gate voltage sweep (Δ*V*_Dirac_) is a measure of the hysteresis window. The large hysteresis window and the high Dirac voltage are mainly attributed to doping effects from both the SiO_2_ substrate and from the residues resulting from the incomplete removal of PMMA^15^. These residues create trapping sites at the surface of the transferred graphene, which results in a degradation of the electrical properties and reduce the reliability of graphene devices^13^. A narrow distribution of the *Dirac voltage* is indicative of both less doping and less trapping in the transferred graphene. For the PMMA/PVA transferred graphene FETs, a more negative shift in the Dirac voltage from −2 to 10 V while the gate voltage was scanned forward and backward, and a clear reduction of the hysteresis window (12 V) were observed (Fig. 3c). This result is a proof of the clean surface and low levels of doping or polymer residues in the transferred graphene. An average Dirac voltage value of 4 V, which is close to the ideal value for graphene FETs (*i.e*. 0 V), and a small hysteresis window was observed for the PMMA/PVA method, which could be due to the inevitable doping effects originated from contamination with ionic or metal residues, to maintain its charge neutrality^14^,^24^. It has been reported that the presence of water molecules at the graphene/SiO_2_ interface enhances the molecular adsorption from the ambient environment resulting in p-doping, high Dirac voltage, gate hysteresis, and reduced mobility in graphene FETs^16^,^25^,^26^. For a complete and consistent analysis, more than 100 graphene FET devices made using both PMMA and PMMA/PVA methods were evaluated; the PMMA/PVA graphene FETs showed small average Dirac voltage (4 ± 5 V, *N* = 140) while a large average Dirac voltage (38 ± 7 V,*N* = 100) was observed for the PMMA graphene FETs (Fig. 3b,d). Further, a large negative shift in the average Dirac voltage from 4 V to 38 V was observed for the PMMA and PMMA/PVA methods, respectively. The effective suppression of hysteresis and low Dirac voltage in the PMMA/PVA graphene FET originates from the absence of charge-trapping sites, *i.e*., polymer residues on the surface of the transferred graphene.

In addition to change in the Dirac voltage and the hysteresis window, the FETs from PMMA/PVA transferred graphene exhibited much enhanced mobility, conductivity and improved symmetry in charge-carrier transport as compared to the PMMA transferred graphene FETs. The mobilities of the n-type and p-type carriers of graphene FETs are also extracted from the *I*_ds_–*V*_g_ transfer characteristics and are shown in Fig. 3a,c. The carrier modulation and the average electron mobility are 1,607 and 3,587 cm^2^ V^−1^ s^−1^ for the PMMA and PMMA/PVA samples, respectively and the hole mobilities are increased from 1,687 in the PMMA sample to 3,800 cm^2^ V^−1^ s^−1^ for the PMMA/PVA graphene FET. Thus, the mobility for both n-type and p-type carriers is enhanced by more than twice for the PMMA/PVA graphene. The mobility value was extracted using the Drude model, which gives the dependence of the mobility on the carrier concentration^27^. The carrier mobility (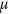) and the carrier density (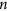) can be calculated from the equation


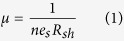



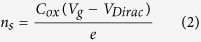


where 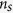 is the variable carrier density, which depends on the back-gate voltage; 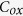 is the gate oxide capacitance (34.5 nF cm^−2^) and *e* is the electronic charge. The sheet resistance (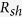) was calculated as


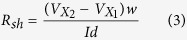


where 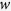 = *10* *μ*m is the channel width, 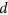 = *10* *μ*m is the distance between the voltage leads 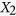 and 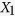, and 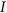 is the current flowing between the leads 1 and 2.

**Figure 4:**
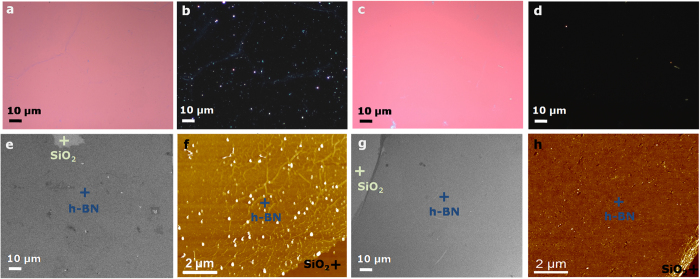
Single layer h-BN transfer. Optical microscopy images of (**a,c**) bright field and (**b,d**) dark field, (**e,g**) SEM images, (**f,h**) Raman spectra and AFM images of single layer h-BN on SiO_2_/Si substrate transferred by bubbling transfer method using PMMA and PMMA/PVA, respectively.

Apart from substrate etching methods, transfer methods based on bubbling have also been used to transfer CVD grown 2D materials such as h-BN layer on Pt foil^28^ and graphene on Cu foil^29^,^30^ onto arbitrary substrates using PMMA assisted transfer. This method avoids metal etching and the 2D material films are transferred by direct delamination from the growth substrate. We also successfully demonstrated a PMMA/PVA assisted transfer of CVD grown single layer h-BN on Pt foil onto an arbitrary substrate by using the bubbling method. The schematic diagram of the PVA assisted transfer of CVD-grown h-BN by bubbling transfer method is shown in Fig. 1b. The detailed transfer procedure is also described in the Experimental Section. Similar to the PVA graphene transfer process, instead of etching the copper foil in step 2, the bubbling method based on an electrochemical reaction was used to release the single layer of h-BN from the Pt foil^28^. The quality of the transferred h-BN on 300 nm SiO_2_/Si substrate was examined in detail by optical microscopy, SEM, AFM and Raman spectroscopy for both PMMA and PMMA/PVA based transfer methods for comparison. As in Fig. 4, optical microscopy images of (a,c) bright field and (b,d) dark field, (e,g) SEM images and (f,h) AFM images of the transferred single layer h-BN on SiO_2_/Si substrate by the bubbling method using PMMA and PMMA/PVA, respectively are shown. From these results, we can conclude that, the PMMA/PVA method leads to a much cleaner sample, almost free from polymer residue when compared to the PMMA method. The single layer h-BN was also characterized by Raman spectroscopy but so far, we did not observe any significant difference in the Raman spectrum between the PMMA and PMMA/PVA transferred h-BN sample (Supplementary Fig. S3). It should noted that in the case of the PMMA/PVA transferred graphene, while no visible polymer residues were observed in the AFM images (Fig. 2g), the optical dark field image (Fig. 2d) revealed the presence of some residual contamination on the surface. These could be either ionic impurities from the metal etchant or metallic residues from the incomplete etching of the metal. These contaminants are trapped at the interface between the transferred graphene and the target substrate, which results in a degradation of the electrical properties and reduces the reliability of graphene devices^14^,^29^. A clean surface free of contamination was observed in the case of PMMA/PVA transferred h-BN as shown in Fig. 4c,d,g,h. We can expect that the bubbling transfer method can therefore lead to better electrical, optical and mechanical properties in devices based on 2D materials. In general, our PMMA/PVA method can be applied to transfer other 2D materials grown on metal substrates such as graphene on copper foil^29^,^30^, MoS_2_ on Au foil^31^ among others.

**Figure 5:**
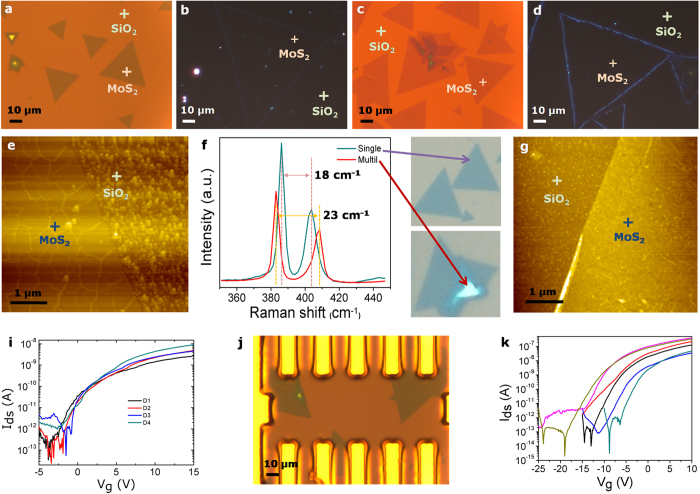
MoS_2_ transfer. Optical microscopy images of (**a,c**) bright field and (**b,d**) dark field, (**e,g**) AFM images, (**f**) Raman spectra; (**i,k**) *I*_ds_–*V*_g_ transfer characteristics and (**j**) optical images of MoS_2_ FETs fabricated from MoS_2_ on SiO_2_/Si substrate transferred by SiO_2_ substrate etching transfer method using PMMA and PVA, respectively.

Finally, the PVA assisted transfer of CVD grown atomic layers of MoS_2_ on 300 nm SiO_2_/Si substrate onto arbitrary substrates was also successfully demonstrated. A schematic diagram of this process involving the etching of the oxide layer is shown in Supplementary Fig. S4. The detailed transfer procedure is described in the Experimental Section. Similar to the PMMA/PVA graphene transfer process, the SiO_2_ oxide layer etching was carried out instead of etching the copper foil in step 2. The quality of the transferred MoS_2_ on 300 nm SiO_2_/Si substrate was carefully examined by optical microscopy, AFM and Raman spectroscopy for both PMMA and PMMA/PVA methods for comparison. From optical, and AFM images (Fig. 5) we can conclude that the PMMA/PVA method helps to transfer clean MoS_2_ layers onto arbitrary substrates without polymer residues. The presence of single and multi-layer MoS_2_ was confirmed by Raman spectroscopy as shown in Fig. 5f,i,k present the transfer characteristics of the drain current versus gate-source voltage (*I*_*ds*_–*V*_*g*_) for several n-type single layer MoS_2_ FETs using MoS_2_ on 100 nm SiO_2_/Si substrate transferred by the conventional PMMA and PMMA/PVA methods, respectively are shown. These were obtained by sweeping the gate voltage continuously from −25 to +15 V with a drain voltage of 0.1 V. The transconductance (*g*_*m*_), the electron mobility (*μ*_*e*_), the subthreshold swing (*S.S*) and the carrier concentration are estimated from the transfer characteristics of the drain current vs the gate-source voltage (*I*_*ds*_–*V*_*g*_). We also observed an enhancement in the device performance. For instance, the transconductance increased from 7.5 to 83 nS, electron mobility increased from 1.1 to 12 cm^2^ V^−1^ s^−1^ while subthreshold swing reduced from 30 to 22 mV dec^−1^ for PMMA and PMMA/PVA-MoS_2_ FETs, respectively (Supplementary S5). These results further confirm that the PMMA/PVA transfer method leads to materials with high quality, low levels of contamination and free from polymer residues. In general, our PMMA/PVA method can be used not only for transferring atomic layers of MoS_2_, but can also be applied to other 2D transition metal dichalcogenide materials grown on oxide substrates onto different types of substrates for high quality, large area, contamination-free layers for many viable device applications.

In summary, we have developed a novel, facile transfer technique for CVD-grown 2D materials. Several 2D materials grown on different rigid substrates such as graphene on copper foil, h-BN on platinum and MoS_2_ on SiO_2_/Si substrates have been transferred using this method. The quality of the transferred materials is carefully examined by optical microscopy, SEM, AFM, Raman spectroscopy and IV characteristics. Graphene and MoS_2_ FET devices from PMMA/PVA transferred layers showed negative shift of Dirac point and higher performance when compared to devices fabricated using the PMMA assisted transfer process. This strategy can be extended to several other 2D material systems grown on different types of substrates. Multilayer stacking of heterostructures of 2D materials can be reliably performed on the wafer-scale, thus paving the way to better optoelectronic, mechanical and nanoelectronic devices.

## Methods

### PMMA/PVA assisted transfer of CVD-grown graphene on copper foil

The monolayer graphene used in this study was grown on a polished copper (111) foil using CVD method, as reported previously by Nguyen *et al*.^32^

A schematic illustration of the transfer process by PMMA/PVA assisted transfer of single-layer graphene is shown in Fig. 1a. The first step of the process involves spin-coating an aqueous PVA solution on CVD-graphene growth substrate (graphene/copper/PET), at step1:1000 rpm for 10 s and step 2:3000 rpm for 60 s, followed by baking at 100 °C for 60 s using a hotplate. Aqueous PVA solution was prepared by dissolving 150 mg of PVA powder in 20 ml DI water and stirring at 120 °C for 2 h. Next, a layer of PMMA was spin-coated on top of the PVA layer, at step1:1000 rpm for 10 s and step2:3000 rpm for 60 s. The PMMA/PVA/graphene/copper foil block was floated on the surface of a solution of 0.3 M ammonium persulfate (Aldrich, ≥98%) at 0 °C for 24 h to etch the copper foil. After the copper was etched, the PMMA/PVA/graphene block was rinsed with deionized water three times at 0 °C and transferred onto a 100 nm thick SiO_2_/Si substrate. Prior to the transfer, the SiO_2_/Si substrate was treated for 5 min in oxygen plasma to render the SiO_2_ substrate hydrophilic and ensure a better wetting of graphene. The transferred substrate was dried under reduced pressure (~10 millitorr) for 24 h and left in air for three days. Finally, the PMMA/PVA carrier was removed by dissolving the PVA in deionized water at 130 °C.

### PMMA/PVA assisted transfer of CVD-grown single layer h-BN on Pt substrate

The single layer h-BN samples used in this study were grown on Pt foil by CVD method, as reported in detail by Kim *et al*.^28^

The bubbling-based transfer method was used to successfully transfer the h-BN layer grown on Pt foil onto arbitrary substrates that allowed the Pt foil to be recycled^28^. Figure 1b shows the schematic of the bubbling-based transfer method, which is based on the electrolysis of water. After the growth of h-BN on Pt foil, the first layer of PVA was coated by spin-coating an aqueous PVA solution on the h-BN/Pt foil followed by a layer of PMMA on top of the PVA layer. Next, the structure composed of PMMA/PVA/h-BN/Pt foil was slowly dipped vertically into a 1 M aqueous solution of NaOH at 0 °C. The PMMA/PVA/h-BN/Pt foil structure was used as the cathode and a piece of bare Pt foil was used as the anode. The application of a constant current for a few min caused the PMMA/PVA/h-BN layer to detach from the Pt foil due to the formation of bubbles upon H_2_ evolution. After the completion of the transfer process, the PMMA/PVA/h-BN layer was rinsed with deionized water at 0 °C three times to remove any remaining NaOH and transferred onto the target substrate. It should be noted that the Pt foil used in the growth of h-BN could be recycled since it was not consumed during the process. The transferred substrate is dried under reduced pressure (~10 millitorr) for 24 h and left in air for three days. Finally, the PMMA/PVA carrier is removed by dissolving the PVA in deionized water at 130 °C.

### PMMA/PVA assisted transfer of CVD-grown single layer MoS_2_ on SiO_2_/Si substrate

The atomic layers of MoS_2_ used in this study were grown on 300 nm SiO_2_/Si substrate by CVD, as reported in detail by Yu *et al*.^33^

After the growth of MoS_2_ on SiO_2_/Si substrate, the first layer of PVA was coated by spin-coating an aqueous PVA solution on the MoS_2_/SiO_2_/Si substrate. The substrate was baked at 100 °C for 1 min on a hotplate. This is followed by coating a second layer of PMMA on top of the PVA layer. The PMMA/PVA/MoS_2_/SiO_2_/Si substrate block was floated on the surface of 1 M Potassium hydroxide solution (Aldrich, ≥98%) at 0 °C for 48 h to etch the SiO_2_. After the etching, the PMMA/PVA/MoS_2_ block was rinsed three times with deionized water at 0 °C and transferred onto a 100 nm thick SiO_2_/Si substrate. Prior to the transfer, the SiO_2_/Si substrates were treated for 5 min in oxygen plasma to render them hydrophilic to ensure better wetting of graphene. The transferred substrate was then dried under reduced pressure (~10 millitorr) for 24 h and left in air for three days. Finally, the PMMA/PVA carrier was removed by dissolving PVA in deionized water at 130 °C.

### Fabrication and measurement of Graphene and MoS_2_ field effect transistors

Graphene and MoS_2_ were transferred onto a silicon substrate using both PMMA and PMMA/PVA methods. A heavily doped p-type Si substrate (0.005 Ωcm) was employed as the back gate with a 100-nm-thick, thermally oxidized SiO_2_ top layer as the gate oxide layer. Multiple electrodes were patterned on the 2D materials by a conventional photolithography process. Subsequently, contact electrodes to form ohmic contacts were deposited by electron-beam evaporation as follows: a 20-nm-thick chromium layer was first evaporated, followed by a 50-nm-thick Au layer. The electrical characteristics of the devices were measured in vacuum using a probe station with a Keithley SCS-4200 system.

## Additional Information

**How to cite this article**: Van Ngoc, H. *et al*. PMMA-Etching-Free Transfer of Wafer-scale Chemical Vapor Deposition Two-dimensional Atomic Crystal by a Water Soluble Polyvinyl Alcohol Polymer Method. *Sci. Rep*. **6**, 33096; doi: 10.1038/srep33096 (2016).

## Supplementary Material

Supplementary Information
