# Supplementary material for: PMMA-Etching-Free Transfer of Wafer-scale Chemical Vapor Deposition Two-dimensional Atomic Crystal by a Water Soluble Polyvinyl Alcohol Polymer Method
Source: Sci Rep. 2016 Sep 12;6:33096. doi: 10.1038/srep33096 (PMC5018882; doi:10.1038/srep33096)
Supplement: Supplementary Information [file srep33096-s1.pdf]

Supplementary Information

PMMA-Etching-Free Transfer of Wafer-scale Chemical Vapor Deposition Two-dimensional Atomic Crystal by a Water Soluble Polyvinyl Alcohol Polymer Method

Huynh Van Ngoc[1], Yongteng Qian[1], Suk Kil Han[2] and Dae Joon Kang[1,*]

[1]Department of Physics and Interdisciplinary Course of Physics and Chemistry, Sungkyunkwan University, 2066, Seobu-Ro, Jangan-Gu, Suwon, Gyeonggi-do 16419, Republic of Korea

[2]Teraleader Inc., 55-8, Techno-11ro, Yuseong-gu, Daejeon 34036, Republic of Korea

1. Electrochemical bubbling method for transfer of h-BN.

The front side of the h-BN film on the Pt foil was covered with PMMA. The Pt foil was immersed in an aqueous solution of 1 M NaOH for electrochemical delamination. The PMMA/PAV/h-BN/Pt foil and a bare Pt foil were employed as cathode and anode, respectively. The bubbling transfer was performed under a constant current of 1 A (corresponding electrolytic voltage of 5 - 12 V) for 1 - 5 min. After peeling the PMMA/PAV/h-BN film off from the Pt foil, it was rinsed with deionized water three times to remove residual NaOH. Finally, it was transferred onto a SiO_2(100 nm)/Si substrate.

* Corresponding author: djkang@skku.edu

2. AFM images of transferred graphene after annealing

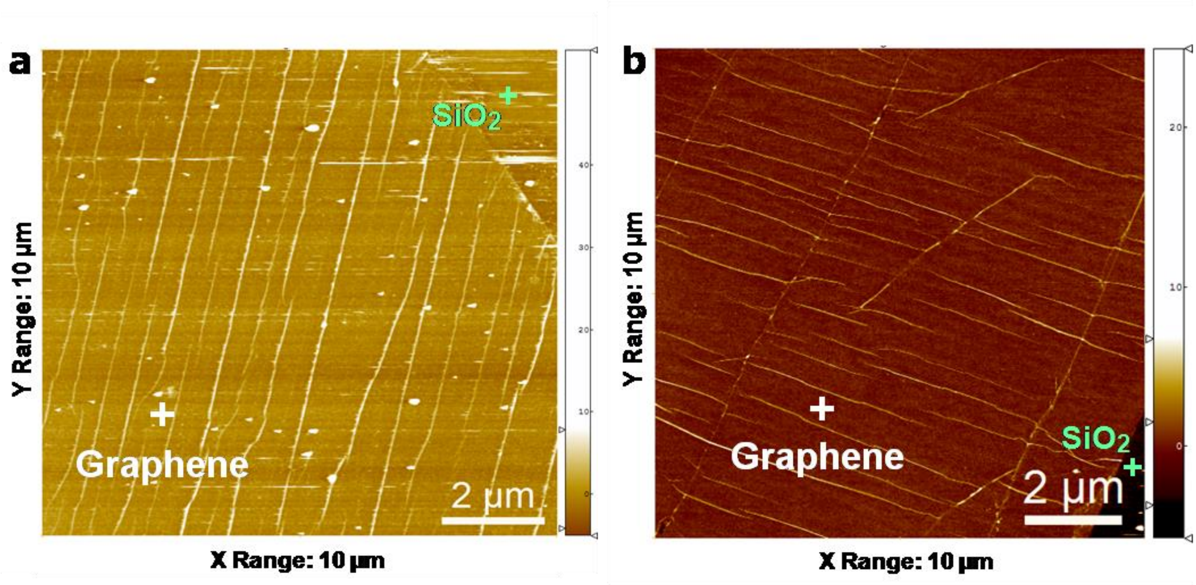

Figure S1. AFM images of graphene on SiO_2/Si substrate transferred using PMMA and PMMMA/PVA, respectively, after annealing.

3. Optical and Raman spectra of transferred graphene after annealing

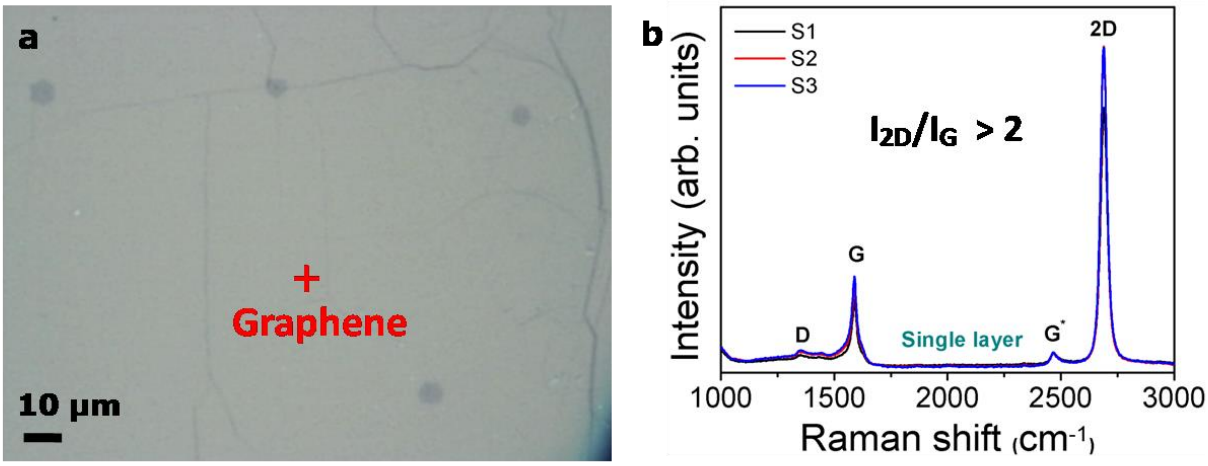

Figure S2. Transferred graphene on SiO_2/Si substrate after annealing using the PMMA/PVA transferring method: (a) Optical microscope image and (b) Raman spectrum

4. Optical images and Raman spectroscopy of single layer h-BN transferred using the PMMA and PMMA/PVA methods

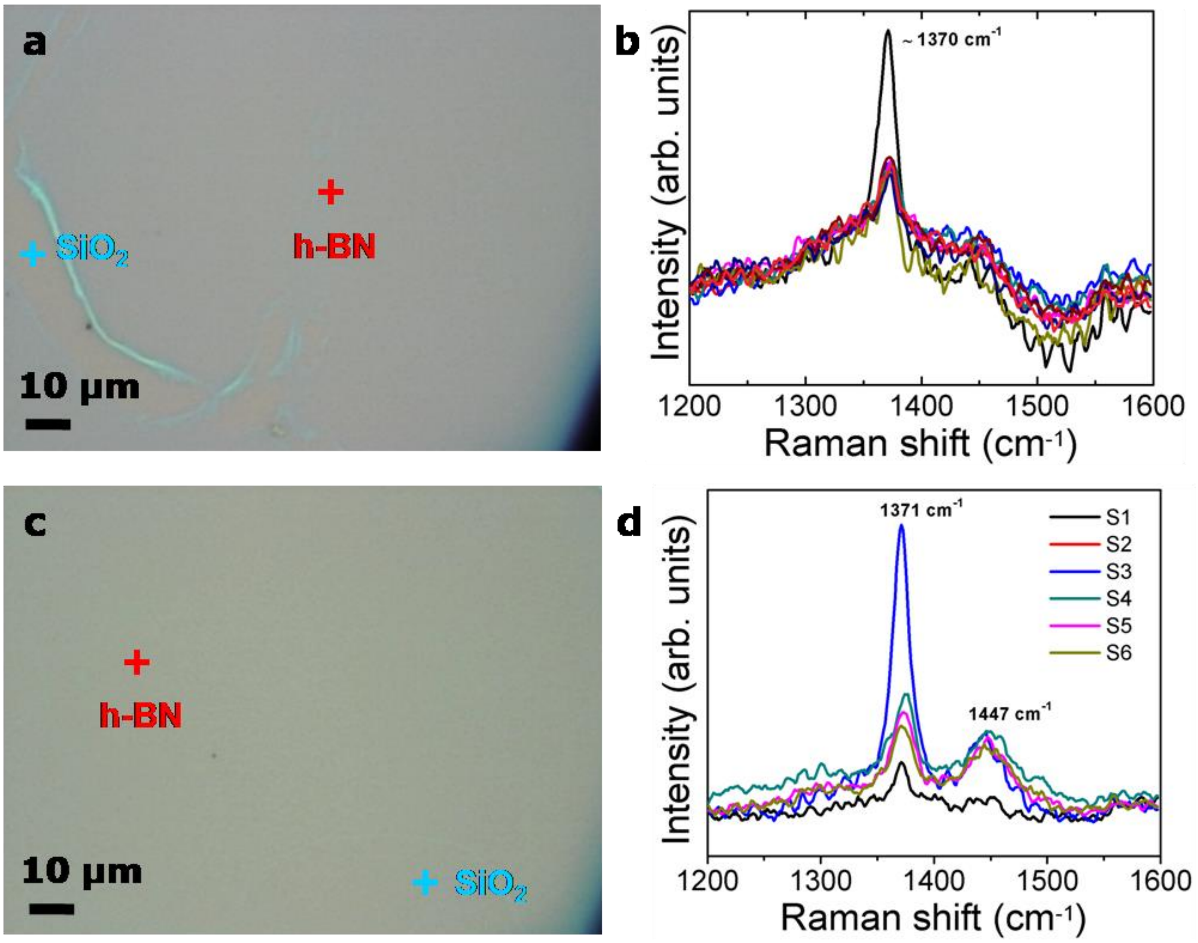

Figure S3. Transferred h-BN on SiO_2/Si substrate: (a, c) Optical microscope image and (b, d) Raman spectroscopy for the PMMA and PMMA/PVA methods of transfer, respectively.

5. Transferring MoS_2 to target substrates

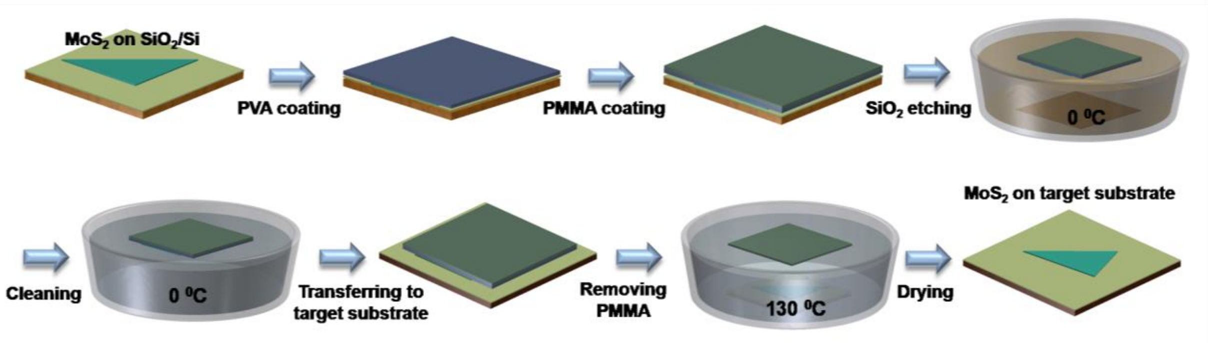

Figure S4. Schematic illustration of PMMA/PVA assisted transfer of CVD-grown MoS_2 onto a SiO_2/Si substrate by oxide layer etching method.

6. Electrical transport properties of single layer MoS_2 FET devices

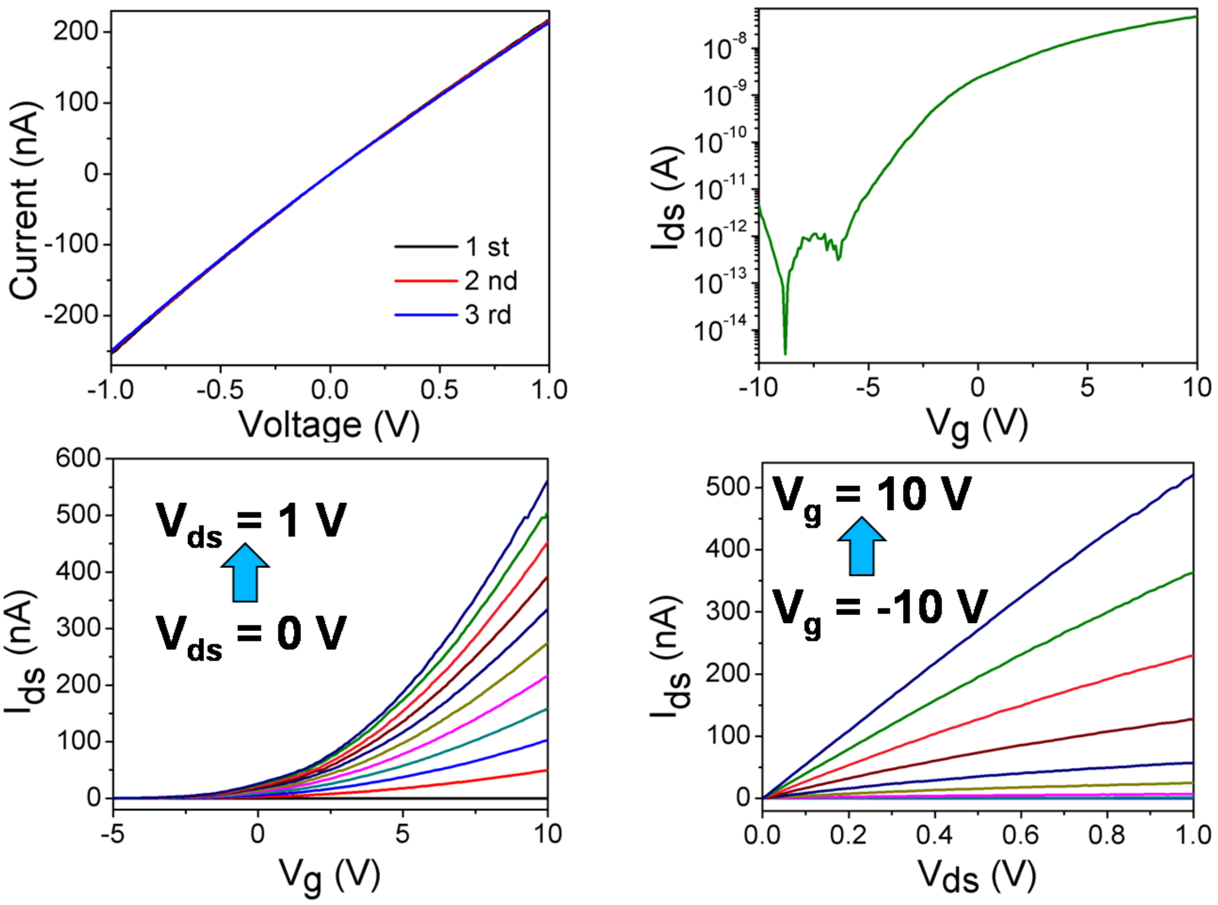

Figure S5. Electrical transport properties at ambient conditions of single layer MoS_2 FET devices based on PMMA/PVA transfer method. (a) Two probe measurements, (b) and (c) I_{ds}–V_g transfer characteristics and (d) I_{ds}–V_{ds} output characteristics.

To investigate the electrical properties of single layer MoS_2, we prepared typical MoS_2 FETs on a 100 nm thick SiO_2 layer on a heavily boron doped Si (p++ Si) substrate. The a-Si served as the back gate electrode. Figure S5d shows the drain current versus drain-source voltage (I_{ds}–V_{ds}) curves for a single layer MoS_2 FET. The conductance of MoS_2 increases monotonically as the gate potential increases from −10 V to +10 V, exhibiting a typical n-type MoS_2 FET behaviour. Figure S5c shows the drain current versus gate-source voltage (I_{ds}-V_g) curves obtained by sweeping the gate voltage continuously from −5 V to 10 V at drain voltages ranging from 0 to 1 V. The transconductance (g_m) and field effect electron mobility (μ_e) of the back gate MoS_2 FETs were determined from the I_{ds}–V_g curves using the following equations: $g_m = dI_{ds}/dV_g$ and $\mu_e = g_m(L/W)(1/V_{ds}C_{ox})$. The gate oxide capacitance (C_{ox}) of a cylindrical wire on a planar substrate can be estimated by the relation $C_{ox} = \varepsilon_r\varepsilon_0 LW/t_{ux}$ using a relative dielectric constant value (ε_r) of 3.9, a SiO_2 gate dielectric layer thickness (t_{ox}) of 100 nm, a MoS_2 conducting channel length (L) of approximately 10 μm, and a width of approximately 20 μm. For n-type MoS_2 FETs on a SiO_2/Si substrate, a threshold voltage (V_{th}) of −6.5 V and a transconductance (g_m) of 83 nS were extrapolated from the linear region of the I_{ds}–V_g curve at a value of 0.1 V for the V_{Ds}. The values of the field-effect electron mobility (μ_e) and the resistivity (ρ) for MoS_2 were calculated from two-probe measurements and found to be 12 $cm^2\ V^{-1}\ s^{-1}$ and 0.21 Ω cm, respectively (Fig. S5a). Electron carrier concentration (n_e) was calculated to be 2.58×10^{17} e/cm^3 using the equation, $n_e = C_{ox}V_{th}/e\pi r^2 L$. The sub-threshold swing given by $S.S = log\ [dV_g/d\ (log I_{ds})]$ was estimated to be 22 mV dec^{-1}.
